# Supplementary material for: Splice-Junction-Based Mapping of Alternative Isoforms in the Human Proteome
Source: Cell Rep. Author manuscript; Available in PMC 2020 Jan 15. (PMC6961840; doi:10.1016/j.celrep.2019.11.026)
Supplement: 3 [file NIHMS1546469-supplement-3.zip › DF2/PXD0010154/Testis-54-Q15435-SQEMMEEEHELPVDMETINLDRDAEDVDLNHYR.pdf]

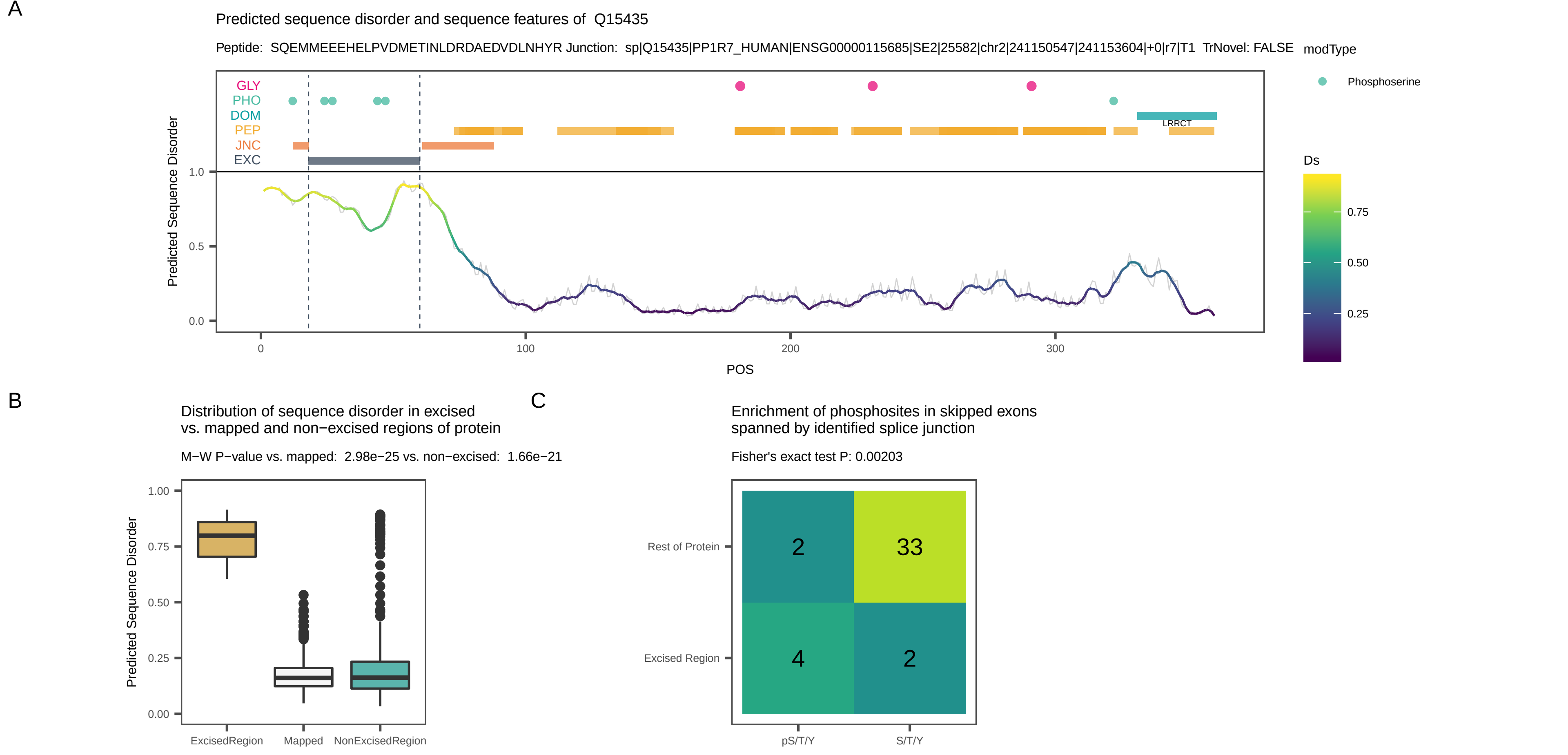

A
Predicted sequence disorder and sequence features of Q15435
Peptide: SQEMMEEEHELPVDMETINLDRDAEDVDLNHYR Junction: sp|Q15435|PP1R7_HUMAN|ENSG00000115685|SE2|25582|chr2|241150547|241153604|+0|r7|T1 TrNovel: FALSE
modType
Phosphoserine
GLY
PHO
DOM
PEP
JNC
EXC
LRRCT
Predicted Sequence Disorder
1.0
0.5
0.0
0
100
200
300
POS
Ds
0.75
0.50
0.25
B
Distribution of sequence disorder in excised vs. mapped and non−excised regions of protein
M−W P−value vs. mapped: 2.98e−25 vs. non−excised: 1.66e−21
Predicted Sequence Disorder
1.00
0.75
0.50
0.25
0.00
ExcisedRegion
Mapped
NonExcisedRegion
C
Enrichment of phosphosites in skipped exons spanned by identified splice junction
Fisher's exact test P: 0.00203
Rest of Protein
Excised Region
2
33
4
2
pS/T/Y
S/T/Y
